# Differences in consonant confusion patterns between bimodal cochlear implant users with greater and less bimodal advantage

**DOI:** 10.3389/fpsyg.2025.1647119

**Published:** 2025-12-10

**Authors:** Yang-Soo Yoon, Amir Majidpour

**Affiliations:** Laboratory of Translational Auditory Research, Department of Communication Sciences and Disorders, Baylor University, Waco, TX, United States

**Keywords:** bimodal listening strategy, spectral integration, spectral interference, ear dominance, bimodal advantage

## Abstract

**Objectives:**

The variability in bimodal advantage among bimodal users remains a significant challenge in clinical approaches. This study compared confusion patterns in consonant recognition between bimodal users who received greater bimodal advantage (GBA) and those who received less bimodal advantage (LBA), aiming to clarify these patterns in order to identify underlying causes that can help improve speech perception for all users.

**Methods:**

Confusion matrices were measured monaurally and binaurally in both quiet and noisy conditions. Twenty-one subjects were divided into GBA (*n* = 8) and LBA (*n* = 13) groups using K-means clustering based on percentage points of bimodal advantage. Participants represented a diverse range of hearing experiences, ensuring the study’s findings would apply to many bimodal users.

**Results:**

By analyzing consonant confusion patterns, the study identifies distinct listening strategies that differentiate GBA and LBA groups. Spectral integration was observed on /ga/, /ma/, /fa/, /ʃa/, /va/, /za/, and /ʤa/ for the GBA group but only on /ga/ and /za/ for the LBA group. Spectral interference was observed on /na/ for the GBA group, but on /ba/, /da/, /pa/, /ta/, /ka/, /ma/, /sa/, and /ʤa/ for the LBA group. Cochlear implant (CI)-ear dominance occurred on /ba/, /da/, /pa/, /ta/, /ka/, and /sa/ for the GBA group and on /na/, /fa/, /ʃa/, and /va/ for the LBA group.

**Conclusion:**

These findings suggest that individual differences in speech perception among bimodal users reflect how each individual combines input from both ears. Three distinct listening strategies emerged: spectral integration, spectral interference, and CI-ear dominance. Users with greater advantage were more successful at integrating complementary inputs across ears, while others experienced conflicting auditory inputs or mainly relied on their CI ear. These results highlight the importance of looking beyond overall speech scores and focusing on specific speech sound patterns to understand why some bimodal users benefit more than others. By identifying the roles of listening strategies in consonant perception, we can better explain individual variability and improve clinical approaches to hearing device fitting.

## 1 Introduction

Bimodal hearing, combined use of a cochlear implant (CI) in one ear and a hearing aid (HA) in the contralateral ear, is a clinical recommendation for individuals with useful residual hearing in the HA ear. Many bimodal users experience a significant bimodal advantage, which is an improved performance with the combined use of a CI and HA compared to using the better device alone in speech perception (Gifford and Dorman, 2019; Yoon et al., 2012a). However, some bimodal users find little to no bimodal advantage (Dorman et al., 2008; Gifford et al., 2013; van Loon et al., 2017). In some cases, bimodal input can result in a bimodal disadvantage, resulting in a lower performance with the combined use of a CI and HA compared to the better device alone (Bernstein et al., 2019; Fowler et al., 2016; Fraysse et al., 2006; Goupell et al., 2018; Neuman et al., 2017). This variability may be due to individual differences in the ability to integrate acoustic and electric information (Jaisinghani and Yoon, 2025), which is essential for receiving rich spectro-temporal information across the two modalities. These spectral integration abilities can be limited by spectral interference (Imsiecke et al., 2020b) and/or better ear dominance (Oh and Reiss, 2017).

In bimodal hearing, the spectro-temporal information is different across ears due to limited residual hearing in the HA ear (Fowler et al., 2016; Neuman et al., 2019; Sheffield et al., 2016; Skarzynski et al., 2013; Zhang et al., 2010) and frequency-to-place mismatch in the CI ear (Ketten et al., 1998). Individual bimodal users require an efficient spectral integration process to achieve optimal bimodal advantage. Spectral integration refers to detecting auditory cues processed by each ear and utilizing both cues to achieve better perception without forming a unified composite. Fu and colleagues explored spectral integration for vowel perception using a simulation of bimodal hearing (Fu et al., 2017a; Fu et al., 2017b). They derived integration efficiency, which is a ratio between the observed and predicted performance of the HA ear and CI ear from raw percent correct scores in the HA ear alone, CI ear alone, and bimodal conditions. However, the integration efficiency does not indicate how specific frequencies independently processed by an HA ear and a CI ear were integrated. Thus, it is unclear whether the bimodal advantage was due to spectral integration or something else. Reiss and colleagues measured spectral integration by determining a dichotic fusion range of pure tones (Oh and Reiss, 2017; Reiss et al., 2018; Reiss et al., 2014). Bimodal users had a wider pitch fusion range (one to four octaves) than listeners with normal hearing (less than 0.2 octaves). However, pitch fusion measurement differs from spectral integration measurement. Pitch fusion refers to blending two independent frequencies evoking different pitches in each ear into a single new pitch percept, which is considered a distortion. Recently, Jaisinghani and Yoon (2025) measured the spectral integration of the first two formant frequencies (F1 and F2) for vowel perception using an acoustic simulation of bimodal hearing. The acoustically simulated F1 was presented to the left ear, while the electrically simulated F2 was presented to the right ear as a function of the frequency spacing between F2 and F1 (i.e., F2 minus F1). Bimodal advantage improved when the frequency spacing was smaller, indicating that bimodal hearing integrates narrower frequency spacing better than wider frequency spacing across ears.

While spectral integration enhances perception by making complementary use of cues from both ears, spectral interference harms perception when auditory cues conflict or are poorly combined, resulting in a bimodal disadvantage. Mok and colleagues observed a reduced bimodal advantage in users with measurable aided thresholds at 4000 Hz in the HA ear (Mok et al., 2010; Mok et al., 2006). Similarly, Fowler et al. (2016) found that bimodal users with better residual hearing (< 60 dB HL at 250 and 500 Hz) showed improved speech perception in quiet when low-to-mid frequency information (approximately 440–982 Hz) from the CI ear was removed, suggesting that eliminating mid-frequency input from the CI reduces spectral interference. Bimodal disadvantage also occurred when the HA-ear alone performance exceeded CI-alone performance (Neuman et al., 2019). They compared two bimodal groups: 21 subjects who performed better with the CI than the HA (CI ear > HA ear) and 10 subjects who performed better with the HA than the CI (HA ear > CI ear) on speech perception tasks in noise. All subjects in the HA > CI group experienced a bimodal disadvantage. Collectively, these findings support the idea that spectral interference between ears can cause bimodal disadvantage – specifically, when finer spectral details available through better residual hearing are negatively affected by mismatched CI-processed spectral information. Recent findings by Jaisinghani and Yoon (2025) further link spectral interference to the ear, providing the dominant cue for recognition. In a bimodal simulation study, they found that 12 out of 20 subjects (60%) showed a 15.5% decrease in identification for the front vowel /I/ under bimodal conditions. They speculated that spectral interference occurs when the CI ear conveys the dominant cue (e.g., F2), while the HA ear provides the non-dominant cue (e.g., F1), an idea also supported by Dubno and Dorman (1987). Moreover, Jaisinghani and Yoon (2025) showed that interference was more likely when F1 and F2 cues were separated by 2.25 octaves, extending previous findings that abnormal fusion and interference occur in bimodal users when pitches are separated by three to four octaves (Oh and Reiss, 2020; Reiss et al., 2014). These results suggest that spectral interference may arise at or beyond the binaural interaction level in the central auditory system due to abnormally broad pitch fusion. It is important to note that not all listeners experience interference: eight subjects in Jaisinghani and Yoon (2025) did not show signs of spectral interference. This listener-specific variability highlights the need to further investigate factors contributing to individual differences in bimodal perception, consistent with the suggestion by Kiefer et al. (2005).

Ear dominance, the tendency for auditory information from one ear to be preferentially processed and perceived over information from the opposite ear, often occurs in bimodal hearing due to asymmetrical inputs across ears, such as differences in frequency, timing, and intensity (Pieper et al., 2022). Reiss et al. (2014) observed CI-ear dominance in a pitch fusion study with bimodal users. Oh and Reiss (2020) found that pitch dominance in bimodal hearing is determined by the ear receiving the lower pitch when two tones were widely separated. Yoon et al. (2015) showed that ear dominance is inversely related to bimodal advantage. Specifically, bimodal users with large performance differences between the CI and HA ears (greater than 30%) experience poorer bimodal advantage, whereas those with smaller performance differences show greater bimodal advantage. In cases of CI-ear dominance, information presented to the CI ear is primarily processed and perceived, while information from the HA ear is less utilized.

One effective way to illustrate how spectral integration, interference, and ear dominance affect consonant recognition is by using confusion matrices. Confusion matrices provide a detailed view of sound perception by showing both correct and incorrect responses (Allen, 2005; Miller and Nicely, 1955). By comparing confusion matrices collected under HA ear alone, CI ear alone, and bimodal hearing conditions, we can identify whether a bimodal user relies more on integration, interference, or CI ear dominance. For example, if there is reduced confusion in the bimodal hearing compared to the better ear alone (typically, the CI ear alone), it suggests better integration. Comparing consonant confusion patterns between groups with greater bimodal advantage (GBA) and less bimodal advantage (LBA) can help determine whether integration contributes to bimodal advantage or disadvantage. The value of confusion matrices in providing detailed information about speech perception errors has been discussed in previous studies. Fu and Galvin III (2008) applied confusion matrix analysis to identify which speech contrasts remained difficult for CI users during training. This approach allowed a more precise evaluation of learning patterns and helped adapt training strategies to target persistent perceptual errors. Confusion matrices provide detailed information about the errors made by CI users when identifying sounds. Chatterjee and Peng (2008) used confusion matrices to examine how CI users responded to pitch-related cues. The matrices helped visualize which speech contrasts were more challenging to detect, especially when changes in modulation were more sensitive. This approach allowed the authors to track specific patterns of error tied to acoustic differences across stimuli. Similarly, Kong and Braida (2011) used confusion matrices to examine how specific features like voicing and manner of articulation affect consonant and vowel recognition. Their study showed that overlapping acoustic and electric stimulation led to redundancy, resulting in little improvement in consonant identification when both devices were used. In contrast, vowel cues were complementary across devices, as both the HA and CI contributed different features (e.g., vowel height), leading to improved vowel recognition. Incerti et al. (2011) also used confusion matrix analysis to examine consonant confusions in bimodal users under various conditions. They found that bimodal hearing enhanced the perception of voicing features in quiet and improved both voicing and matter cues in noise. Their results showed that using both the CI and HA improved the transmission of phonetic features (voicing, manner, and sibilance) compared to using the CI alone. Overall, the confusion matrices were a powerful tool for evaluating spectral integration, interference, and ear dominance in consonant recognition and for providing detailed insights into the nature of advantages and disadvantages in bimodal hearing.

In the present study, we aim to elucidate the intricacies of bimodal hearing by analyzing consonant confusion matrices and comparing the GBA and LBA groups. Two main questions were asked: (1) Which consonant contributed to differentiating the GBA and LBA groups? (2) Were the confusion patterns different between the groups? If yes, we should consider the reason behind each pattern (Integration, Interference, and CI ear dominance) separately and know which consonants are affected by each pattern. Answering these questions will better explain the differences between bimodal patients with greater and less bimodal advantage in consonant recognition.

## 2 Materials and methods

### 2.1 Subjects

**Figure 1:**
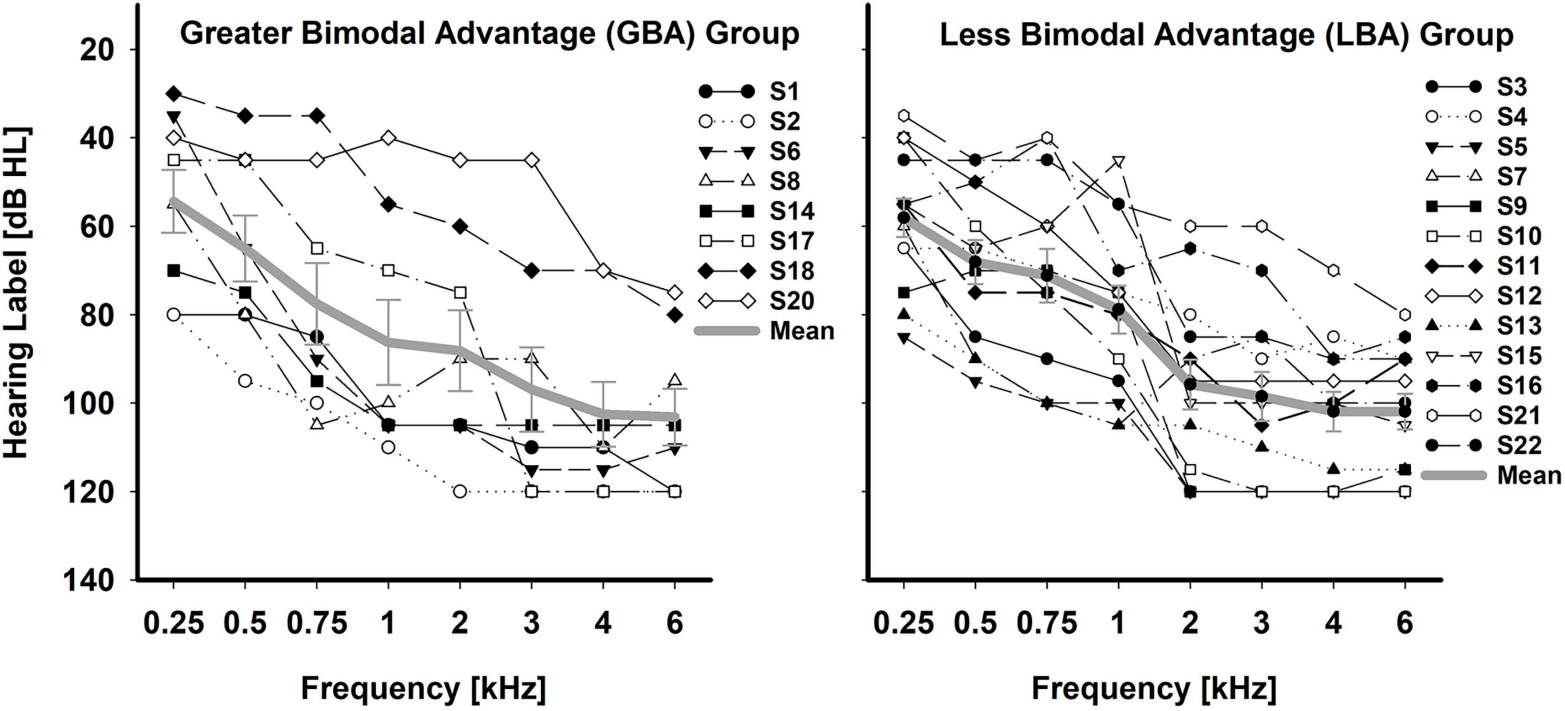
Individual and mean unaided hearing thresholds in the HA ear for the GBA (*n* = 8) group and LBA (*n* = 13) group. The error bar indicates the standard error of the mean.

Twenty-two adult bimodal listeners (12 females and 10 males) participated in the study. However, subject S19 (female, 47 years old) could not complete the entire testing protocol; her data were excluded from the analysis. All 21 subjects were native speakers of American English and were post-lingually deafened. The mean age and standard deviation of subjects at testing were 60.7 ± 11.6 years (38–94 years). The subjects were divided into the GBA (*n* = 8, 59.4 ± 12.5 years) and LBA (*n* = 13, 61.50 ± 11.5 years) groups, based on K-means clustering analysis with percentage points of bimodal advantage (see A. Determination of the GBA and LBA groups in bimodal advantage in the Results section for details). All subjects used both a HA and a CI full-time for at least more than one year (Buchman et al., 2020). Subjects were recruited with no specific configurations and degrees of residual hearing in the HA ear. Individual and group mean unaided hearing thresholds for HA ears were provided on the left panel of Figure 1 for the GBA group and on the right panel of Figure 1 for the LBA group. GBA and LBA groups’ demographic information, along with unaided pure-tone average (PTA) thresholds over 250, 500, 1,000, and 2000 Hz, are shown in Tables 1, 2, respectively. Twelve out of the 21 subjects had equal to or less than three years of experience with their bimodal fittings, two subjects had between three and six years of experience, and seven subjects had more than six years of experience. Due to the inherent right-ear advantage, whereby words reaching the right ear are directly processed in the left hemisphere, where speech perception occurs, without necessitating interhemispheric connections (Hertrich et al., 2002), the orientation of HAs or CIs toward a specific ear was examined to ensure it did not skew the results. It was observed that the participants in the study were evenly distributed between the two groups, ensuring homogeneity. Among the 8 cases in the GBA group, 3 had cochlear implants in the right ear and 5 in the left ear. Similarly, in the LBA group comprising 13 individuals, 7 had CIs in the right ear, while 6 had them in the left ear. Informed consent was obtained from all subjects, and the local Institutional Review Board approved all procedures.

**Table 1:** Greater bimodal advantage (GBA) groups’ demographics, devices, and presentation levels.

ID	Etiology	PTA in HA Ear [dB HL]	CI (L/R ear): Processor, years of use	Years of bimodal hearing	Presentation Level [dBA]
(Age, Gender)			HA (L/R ear): Device, years of use		
S1		93	CI (R): Nucleus 5, 11		CI alone: 70
(75, M)	Aging		HA (L): Phonak Audéo Lumity, 34	11	HA alone: 80 CI + HA: 70
S2		101	CI (L): Nucleus 7, 3		CI alone:70
(47, F)	Unknown		HA (R): Phonak Naida Paradise, 43	3	HA alone: 85 CI + HA: 75
S6		78	CI (L): Nucleus 7, 2		CI alone: 65
(38, M)	Noise-induced		HA (R): Phonak Naida Paradise, 6	2	HA alone: 85 CI + HA: 75
S8		81	CI (R): Nucleus 7, 3	3	CI alone: 65
(62, M)	Unknown		HA (L): Unitron Moxi V-RS, 50		HA alone: 80 CI + HA: 70
S14	Unknown	89	CI (L): Nucleus 6, 7	7	CI alone: 65
(61, F)			HA (R): ReSound Omnia, 50		HA alone: 85 CI + HA: 75
S17		59	CI (L): Nucleus 5, 10	10	CI alone: 65
(54, F)	Noise-induced		HA (R): Starkey 400, 25		HA alone: 85 CI + HA: 65
S18		45	CI (L): Nucleus 6, 7	7	CI alone: 70
(66, M)	Noise-induced		HA (R): Oticon, TEIC, 10		HA alone: 75 CI + HA: 70
S20		43	CI (R): Nucleus 5, 12	12	CI alone: 75
(72, M)	Noise-induced		HA (L): Widex RIC, 20		HA alone: 70 CI + HA: 70

The unaided pure-tone average (PTA) threshold in the HA ear was computed over 250, 500, 1000, and 2000 Hz. L and R indicate the left and right ear.

**Table 2:** Less bimodal advantage (LBA) groups’ subject demographics, devices, and presentation levels.

ID (Age, Gender)	Etiology	PTA in HA Ear [dB HL]	CI (L/R ear): Processor, years of use	Years of bimodal hearing	Presentation Level [dBA]
			HA (L/R ear): Device, years of use		
S3		91	CI (R): Opus 2, 11		CI alone:65
(68, F)	Unknown		HA (L): Widex Senso Diva, 20	11	HA alone: 80 CI + HA: 75
S4		71	CI (L): Nucleus 8, 1	1	CI alone:65
(74, F)	Aging		HA (R): ReSound Nexia, 25		HA alone: 80 CI + HA: 75
S5		100	CI (R): Nucleus 7, 3	3	CI alone: 65
(61, F)	Otosclerosis		HA (L): ReSound Omnia, 25		HA alone: 86 CI + HA: 75
S7	Ototoxicity	86	CI (L): Sonnet 2, 2	2	CI alone: 70
(66, M)			HA (R): Oticon More, 18		HA alone: 80 CI + HA: 70
S9		85	CI (L): Nucleus 7, 2	2	CI alone: 65
(54, M)	Unknown		HA (R): ReSound Enzo Q, 32		HA alone: 80 CI + HA: 70
S10		76	CI (R): Nucleus 6, 5	5	CI alone: 65
(65, F)	Hereditary		HA (L): Resound Metrix, 37		HA alone: 80 CI + HA: 70
S11		75	CI (R): Naida Q90, 3	3	CI alone: 65
(84, F)	Aging		HA (L): Phonak Virto Paradise, 30		HA alone: 80 CI + HA: 70
S12		65	CI (L): Naida Q90, 2	2	CI alone: 70
(64, M)	Hereditary		HA (R): Phonak Virto Paradise, 19		HA alone: 80 CI + HA: 75
S13		95	CI (R): Nucleus 6, 4.5	4.5	CI alone: 65
(44, F)	Unknown		HA (L): Signia Pure 312 AX, 22		HA alone: 85 CI + HA: 75
S15	Ototoxicity	66	CI (R): Nucleus 5, 7	7	CI alone: 80
(52, M)			HA (L): Oticon Dual Wx Rite, 12		HA alone: 85 CI + HA: 85
S16		60	CI (R): Nucleus 7, 2	2	CI alone: 65
(48, M)	Unknown		HA (L): Oticon Zircon, 5		HA alone: 80 CI + HA: 65
S21		49	CI (L): Naida Q90, 2	2	CI alone: 70
(70, F)	Noise-induced		HA (R): Phonak Audéo Lumity, 15		HA alone: 75 CI + HA: 70
S22	Unknown	58	CI (L): Nucleus 6, 7	7	CI alone: 60
(50, F)			HA (R): Phonak Naida, 12		HA alone: 75 CI + HA: 60

Unaided PTA threshold in the HA ear was computed over 250, 500, 1000, and 2000 Hz. L and R indicate the left and right ear.

### 2.2 Stimuli

Fourteen American English consonants with the common vowel /a/ as in “father” [/b/, /d/, /g/, /p/, /t/, /k/, /m/, /n/, /f/, /s/, /ʃ/, /v/, /z/, and /ʤ/] were used as stimuli (Fousek et al., 2004). These consonants were selected because they are the most frequently used consonants in American English (Hayden, 1950). An affricate consonant (/ʤ/) was not considered one of the most commonly used consonants, but was included because it is often confused with /b/, /g/, /k/, and /t/ in CI users (Rødvik et al., 2019). Consonant-vowel (CV) syllables were spoken by five male talkers (mean fundamental frequency: 115 Hz), resulting in 70 tokens in total. The mean duration of syllables was 406.6 ms with a standard deviation of 106.6 ms.

Consonant recognition testing was measured in quiet and noise at 5 dB and 10 dB signal-to-noise ratio (SNR). The decision to use 5 dB and 10 dB SNRs was based on our preliminary studies with bimodal users, which demonstrated a wide range of performance (30 to 70%) under these SNRs, avoiding floor and ceiling effects while allowing room for improvement (Yoon et al., 2019a). The noise level was adjusted relative to a fixed speech level to achieve the SNRs. Speech-shaped noise was generated by combining the long-term average spectrum of concatenated speech from 70 CV syllables with white noise (duration: 2 s, sampling frequency: 44,100 Hz using Praat software) (Boersma, 2001). This noise was added to the syllables to generate the designated SNRs. Speech-shaped noise was used because the information needed to identify individual phonemes occurs over a short time frame, and fluctuations in maskers could lead to performance variability. The sum of the speech signal and masking noise was filtered with a band-pass filter of 100 Hz-8000 Hz before presentation to equalize the bandwidth. The output levels of all CV syllables were normalized by 20 dB down from a reference (i.e., 0 dB full-scale RMS) to avoid signal clipping and ensure the same RMS value for all CV syllables. The overall presentation level of the final stimulus was adjusted to the subject’s most comfortable level in dB(A) SPL, measured for each listening condition (left ear alone, right ear alone, and both ears) as shown in Tables 1, 2. The most comfortable level was determined using 14 CV syllables from the stimuli and 5 dB increments according to the Cox loudness rating scale (Cox, 1995). CV syllables and noise maskers were presented in front of subjects at 0° azimuth to control for potential benefits from binaural squelch and head shadow. During each trial of speech tests, the masker began 500 ms before the onset of the target speech and continued for 500 ms after the target offset, with cosine onset and offset ramps of 100 ms applied.

### 2.3 Procedure

Consonant recognition was measured using a 14-alternative forced-choice paradigm with the subject’s clinical devices for HA alone, CI alone, and bimodal listening conditions at a fixed +5 dB and +10 dB SNR and in quiet conditions. Before testing, a visual inspection was performed on the device. No adjustments were made to the CI or HA settings. Each subject was evaluated using their own clinically fitted, everyday listening programs to best reflect real-world use rather than optimized laboratory conditions. The ear canal was otoscopically inspected, and pure-tone audiometry was performed if the patient’s hearing threshold record was more than one year old. Subjects were seated directly in front of the loudspeaker in a single-walled, sound-treated booth (Industrial Acoustics Company). The loudspeaker (RadioEar SP90; frequency response: 125–8,000 Hz with a sensitivity of 94 dB/W; total harmonic distortion is < 1% at 1 W and < 5% at rated power) was positioned at 0° azimuth and one meter away from the head of the subject. The hearing device on the non-tested ear was off and removed, and the non–tested ear was occluded with a foam hearing protective device (i.e., a single-sided earplug). During bimodal testing, a foam earbud was also placed in the implanted ear to acoustically isolate it. We intentionally chose not to use a direct audio input for both HA and CI to maintain a consistent listening environment and avoid potential confounding variables associated with the varying features of direct audio input with different models of HA and CI. Additionally, we randomized the order of the CV syllables, listening conditions, and SNRs to further minimize the effects of any sequence-related confounders, ensuring the reliability of the results. Before testing, subjects were provided 10 min of familiarization for each listening condition (total of 30 min) in quiet with feedback. The individual stimuli [14 CVs × male 5 talkers × 3 SNRs = 210] were repeated two, four, or six times depending on the subject’s availability, yielding either 10, 20, or 30 repetitions of each CV at each SNR (i.e., row sum of confusion matrix was 10, 20, or 30). A row sum of 10 would be sufficient to generate reliable perceptual confusion (Allen, 2005; Miller and Nicely, 1955). During testing, subjects were instructed to identify the CV syllables presented by selecting the corresponding CV-labeled button on the graphical user interface using a computer mouse. Both pause and repeat buttons were available to the listeners so that they could control the rate at which the stimulus was presented and could repeat the same stimulus up to three times before responding. This approach was taken because research suggests that listeners with hearing loss show no distinct influence of target repetition on performance (Phatak et al., 2009). Subjects were instructed to select the CV syllables they heard or make their best guess if they were unsure. No trial-by-trial feedback was provided during testing. The complete testing protocol, including consent, pure-tone audiometry, familiarization, test, and breaks, took approximately 6 h per subject, requiring two separate visits.

### 2.4 Data analysis

**Figure 2:**
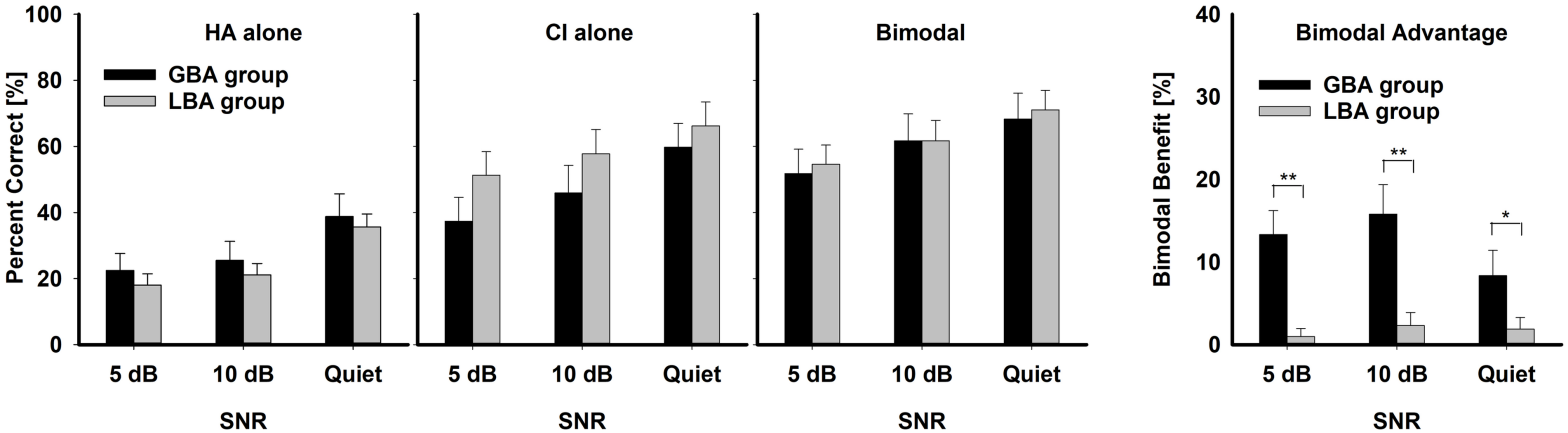
Mean percent correct scores with standard error for HA ear alone, CI alone, and bimodal listening condition for the GBA and LBA groups, along with mean bimodal advantage (most right panel with y-axis scale from 0 to 40 percentage points) for the two groups as a function of SNR. Asterisks indicate the group differences. ** indicates *p* < 0.01 and * *p* < 0.05.

To compare the mean differences between the GBA (*n* = 8) and LBA (*n* = 13) groups for HA alone, CI alone, and bimodal conditions (three left panels of Figure 2), a three-way repeated measures analysis of variance (RM ANOVA) was performed with two within-subject factors (listening condition and SNR) and one between-subject factor (group). To compare the mean bimodal advantage between GBA and LBA groups (the most right panel of Figure 2), a two-way RM ANOVA was performed with one within-subject factor (SNR) and one between-subject factor (group). A two-way RM ANOVA was also performed with group and SNR factors to determine which consonant significantly contributed to the GBA and LBA groups. The results of all statistical analyses were assessed against an alpha level of 0.05 with a two-tailed test. Planned multiple comparisons were performed using an overall alpha level of 0.05 with the Bonferroni correction. Statistical significance was indicated by asterisks: *** for *p* < 0.001, ** for *p* < 0.01, and * for *p* < 0.05.

## 3 Results

### 3.1 Determination of the GBA and LBA groups in bimodal advantage

The range of bimodal advantage in speech perception varies greatly among bimodal users due to several potential factors, such as degrees of residual hearing in the HA ear (Mok et al., 2010; Mok et al., 2006), age (Pillsbury et al., 2018), SNRs (Yoon et al., 2015), and speech stimuli (Gifford et al., 2007). Thus, it is impossible to set a definitive cutoff for a bimodal benefit percentage point to categorize GBA and LBA groups. A K-means cluster analysis was performed to group 21 subjects based on Z-scores obtained from the percentage point benefits. K-means cluster analysis can yield accurate results with z-score normalized data compared to using raw data (Wongoutong, 2024; Zhu et al., 2021). The analysis was conducted with two predefined clusters. The initial cluster centers were −0.89, −0.70, and −0.89 for Cluster 1 and 2.52, 2.75, and 2.80 for Cluster 2. Convergence was reached after two iterations. The final cluster centers were −0.23, −0.12, and −0.22 for Cluster 1 and 2.13, 1.16, and 2.18 for Cluster 2, with a distance of 3.60 between the final cluster centers, indicating a clear separation between the two clusters. ANOVA results showed significant differences between the clusters for all benefits at 5 dB SNR [*F*(1,19) = 20.86, *p* < 0.001] and in quiet conditions [*F*(1,19) = 19.10, *p* < 0.001] but no significant differences at 10 dB SNR [*F*(1,19) = 3.31, *p* = 0.08]. The final solution placed 13 subjects in Cluster 1 (LBA) and eight subjects in Cluster 2 (GBA), as shown in Tables 1, 2, with no missing data.

### 3.2 Mean performance comparisons between the GBA and LBA groups

The left three panels of Figure 2 show the mean percent correct scores of the two groups for each listening condition as a function of SNR. The overall mean percent-correct scores, computed from the row sums of the confusion matrices and averaged across all listening conditions and SNRs, were lower for the GBA group (45.75 ± 2.44%) than for the LBA group (48.61 ± 1.92%), but a three-way RM ANOVA showed that this group difference was not significant, *F*(1,19) = 0.15, *p* = 0.71. In contrast, mean differences across the listening condition were significant, *F*(2,38) = 31.28, *p* = 0.001. Bimodal condition (61.54 ± 6.54) had a higher bimodal advantage than either CI alone (53.07 ± 6.54) or HA alone (26.94 ± 6.54). Significant pair-wise multiple comparison results for the listening condition are given in Table 3. It should be noted that the GBA group had lower scores than the LBA group in CI alone and bimodal conditions. This result is fully discussed in the Discussion section. SNR was also a significant factor in consonant recognition, *F*(2,38) = 135.84, *p* = 0.001. Mean percent correct scores were 56.63 ± 6.54 in quiet, 45.64 ± 6.54 at 10 dB SNR, and 39.27 ± 6.54 at 5 dB SNR. Significant pair-wise multiple comparison results for the SNR variable are given in Table 4. Interactions between listening condition and SNR were significant, *F*(4,76) = 2.56, *p* = 0.04, but other three interactions were not significant: group x listening condition, *F*(2,38) = 1.33, *p* = 0.28; group x SNR, *F*(2,38) = 0.55, *p* = 0.58; group x SNR x listening condition, *F*(4,95) = 1.51, *p* = 0.21.

**Table 3:** Presents significant multiple comparison results for the listening condition within each SNR.

SNR	HA alone vs. CI alone	HA alone vs. Bimodal	CI alone vs. Bimodal
At 5 dB	**	***	**
At 10 dB	***	***	**
In Quiet	**	***	*

*** indicates *p* < 0.001, ** *p* < 0.01, and * *p* < 0.05.

**Table 4:** Presents significant multiple comparison results for the SNR within each listening condition.

Listening condition	5 dB vs. 10 dB	5 dB vs. Quiet	10 dB vs. Quiet
HA alone		***	***
CI alone	***	***	***
Bimodal	***	***	***

*** indicates *p* < 0.001.

The right-most panel of Figure 2 illustrates the comparisons between the two groups on bimodal advantage (percentage point difference between the bimodal listening condition and the better ear alone). The mean bimodal advantage of the GBA group (12.51 ± 8.98%) was statistically significantly higher than that of the LBA group (1.45 ± 4.23%), *F*(1,7) = 14.11, *p* = 0.007. The significant pair-wise multiple comparison results are indicated by asterisks. However, the bimodal advantage was not significantly affected by SNR, *F*(2,14) = 0.77, *p* = 0.48. The mean bimodal advantages were 7.26 ± 6.13 at 5 dB, 7.95 ± 6.61 at 10 dB, and 5.72 ± 7.08 in quiet. Interaction between the group and SNR was also not significant, *F*(2,14) = 2.68, *p* = 0.10.

### 3.3 Comparisons in confusion patterns between the GBA and LBA groups

**Figure 3:**
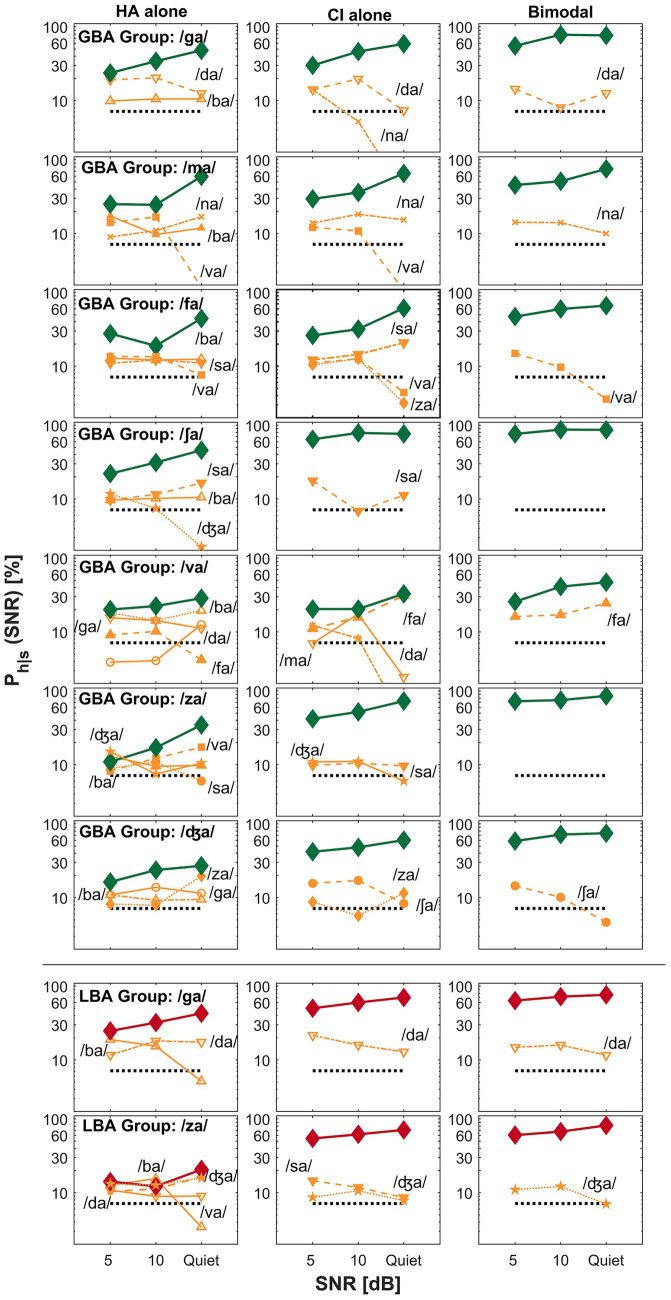
Examples of spectral integration for the GBA group (top seven rows) and LBA group (the bottom two rows) are shown. The left, middle, and right panels represent an HA alone, a CI alone, and a bimodal listening condition, respectively. The ordinate uses a logarithmic scale to better visualize the confusion patterns. The percent correct scores for the presented consonant are denoted as thick lines, whereas competitors or confused consonants are indicated as thin lines with labels. The label *P_h_|_s_* on the y-axis denotes the probability of hearing the intended consonant *h,* given the presented stimulus *s*.

**Figure 4:**
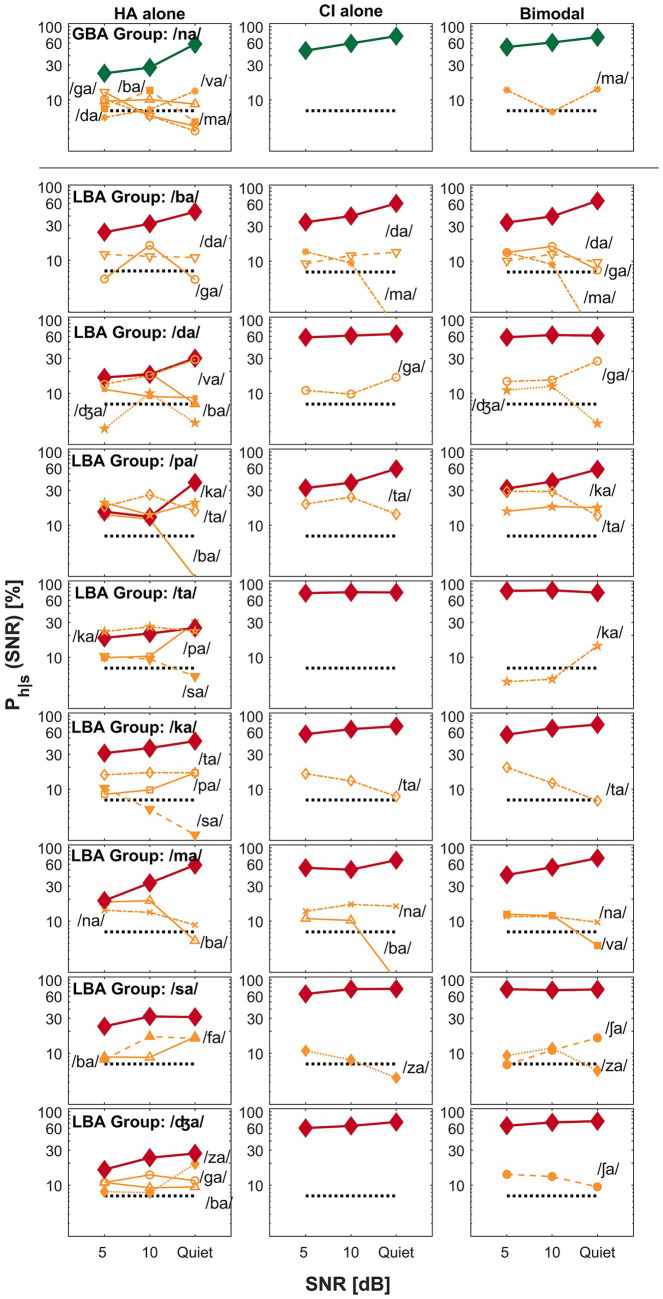
Examples of spectral interference for the GBA group (top row) and the LBA group (remaining eight rows). The panel layout and visualization follow the same format as in Figure 3.

**Figure 5:**
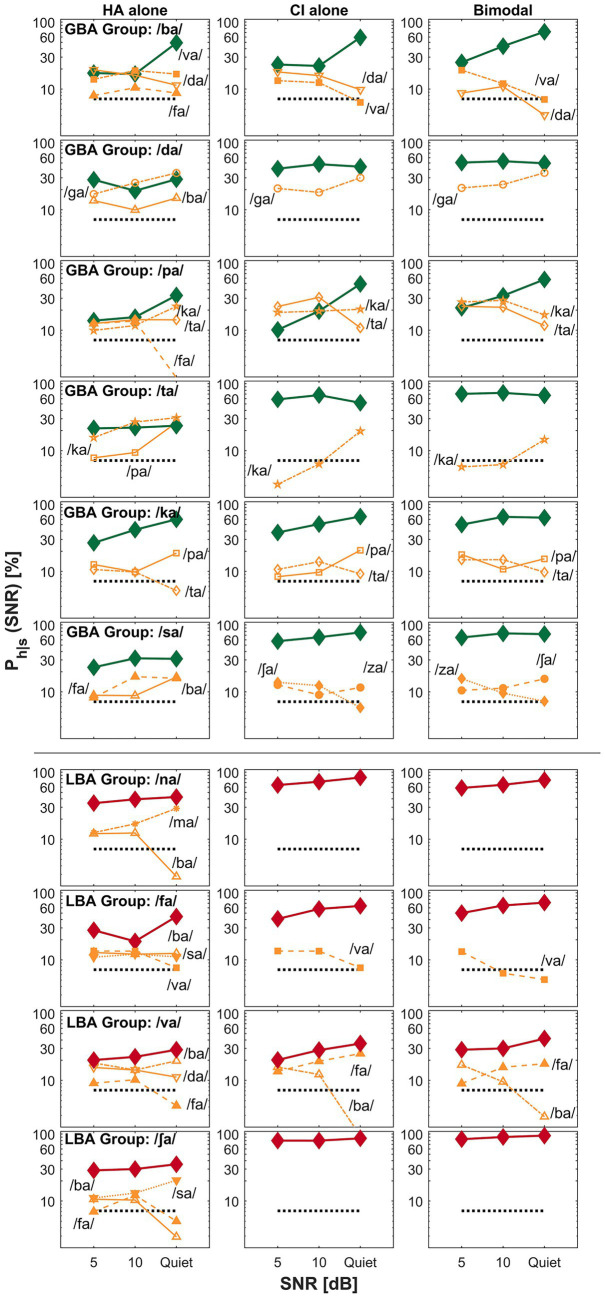
Examples of CI-ear dominance for the GBA group (the top six rows) and the LBA group (the bottom four rows). The panel layout and visualization are consistent with Figure 3.

The primary goal of this current study was to find key differences between GBA and LBA groups by comparing perceptual confusion. In this confusion analysis, we attempted to answer the second research question: were confusion patterns different between the groups? Specifically, we attempted to find how confusion pattern differences are related to spectral integration, spectral interference, or CI-ear dominance. Based on the mean bimodal advantage per CV syllables, confusion patterns of the two exemplary CV syllables (each from the GBA and LBA group) were presented for spectral integration (Figure 3), spectral interference (Figure 4), and CI ear dominance (Figure 5). The raw confusion matrices per subject and per group, are provided in the Supplementary material. For all confusion pattern figures, the left, middle, and right panels indicate an HA alone, a CI alone, and a bimodal listening condition, respectively. The ordinate is a logarithmic scale for the better visualization of confusion patterns. The percent correct scores for the presented CV syllables are denoted as thick lines with symbols. In contrast, the percentage scores for each confused consonant or competitor are indicated as thin lines with symbols and labels. Only up to the top five competitors are shown to avoid overcrowding. The dotted horizontal line indicates a chance level (1/14 * 100 = 7.14%).

#### 3.3.1 Spectral integration

Figure 3 presents examples of spectral integration for nine target CV syllables —shown in the top seven rows for the GBA group and the bottom two rows for the LBA group. Spectral integration refers to the process by which auditory cues from each ear are detected and utilized together to improve perception, without necessarily forming a unified acoustic image. In bimodal hearing, spectral integration is evidenced by reduced perceptual confusions in the bimodal condition compared to those observed in either the HA alone or CI alone condition. The group designation and target CV syllables are listed in the first column of the figure. For the GBA group, seven CV syllables —/ga/, /ma/, /fa/, /ʃa/, /va/, /za/, and /ʤa/—fall into the spectral integration category. For the LBA group, only two CV syllables —/ga/ and /za/—exhibited patterns consistent with spectral integration.

A specific example of spectral integration in the GBA group is illustrated by the confusion patterns when the target CV syllable /za/ (the sixth row from the top) was presented. In the HA alone condition (left panel), four competing CV syllables —/ba/, /sa/, /va/, and /ʤa/—accounted for over 35% of errors at 5 dB SNR and continued to cause more than 25% errors at 10 dB SNR. Notably, confusion with /va/ increased as the SNR improved. In contrast, the CI alone condition (middle panel) showed only two competitors—/ʤa/ and /sa/—which resulted in improved percent correct scores for /za/ across SNRs. Importantly, two of the HA alone competitors—/ba/ and /va/—were not observed in the CI alone condition. In the bimodal condition (right panel), perception of /za/ improved relative to both the HA alone and CI alone conditions. This improvement was due to the resolution of confusions observed in each ear alone, suggesting that the listener successfully integrated complementary CV syllables information from both ears. Based on these patterns, the recognition enhancement of /za/ in the bimodal condition reflects effective spectral integration. Similar patterns were observed for the other six CV syllables in this category.

The bottom two rows of Figure 3 show examples of spectral integration for the LBA group, specifically for the target CV syllables /ga/ and /za/. For /ga/ (the second row from the bottom), two competitors—/ba/ and /da/—were observed in the HA alone condition (left panel). In the CI alone condition (middle panel), only /da/ continued to compete with the target. Under bimodal listening conditions (right panel), the confusion from /da/ was not completely eliminated but was reduced, particularly at 5 dB SNR, leading to improved perception of the target /ga/. A similar pattern was observed for the /za/ target. Confusions present in either the HA alone or CI alone condition were further reduced or resolved in the bimodal condition, resulting in enhanced recognition of /za/. These results demonstrate that even in the LBA group, spectral integration contributed to improved speech perception for select CV syllables.

#### 3.3.2 Spectral interference

While spectral integration enhances perception by combining complementary auditory cues from both ears, spectral interference degrades perception when conflicting or mismatched cues are poorly integrated, resulting in a bimodal disadvantage. In bimodal hearing, spectral interference arises when inputs from the HA and CI conflict, increasing phonetic competition and making speech recognition more difficult than with either device alone. In the current study, the CV syllable /na/ is the only sound categorized under spectral interference for the GBA group, whereas eight CV syllables—/ba/, /da/, /pa/, /ta/, /ka/, /ma/, /sa/, and /ʤa/—fall into this category for the LBA group. The top row of Figure 4 illustrates spectral interference in the GBA group for the target CV syllable /na/. In the HA alone condition, five competing sounds—/ba/, /da/, /ga/, /ma/, and /va/—accounted for over 35% of errors at 5 dB SNR, over 30% at 10 dB SNR, and 25% in quiet. In contrast, the CI alone condition showed no significant competitors. However, in the bimodal condition, the target /na/ experienced a decline in percent correct scores at both 5 dB SNR and in quiet, primarily due to increased confusion with /ma/, demonstrating spectral interference.

For the LBA group, the bottom eight rows of Figure 4 depict spectral interference for the eight CV syllables. For instance, when /ma/ was the target, two competitors—/ba/ and /na/—were present across all listening conditions and SNRs. Importantly, a new competitor, /va/, emerged in the bimodal condition, further reducing the correct perception of /ma/. Similar patterns of interference were observed for the remaining seven CV syllables. In summary, spectral interference can introduce perceptual confusions and lead to a bimodal disadvantage in certain listening contexts.

#### 3.3.3 CI ear dominance

The third category of bimodal listening strategy is ear dominance —the tendency for auditory information from one ear to be preferentially processed and perceived over input from the opposite ear. In bimodal hearing, CI ear dominance typically occurs when the brain relies more on the CI ear than the HA ear for speech understanding. This occurs due to asymmetrical inputs across ears (typically better speech information from the CI ear), making the input from the implanted ear more useful or easier to process than that from the HA. The top six rows of Figure 5 show confusion patterns for the GBA group, where six CV syllables (/ba/, /da/, /pa/, /ta/, /ka/, and /sa/) fall into the CI ear dominance category. For example, when /ba/ (the first row from the top) is the target, it is often confused with /va/, /da/, and /fa/ in the HA ear, while /da/ and /va/ are the main confusions in both the CI ear and bimodal conditions. These similar confusion patterns lead to comparable percent correct scores between the CI alone and bimodal conditions. Similar observations were made for the other five target CV syllables, indicating that confusions in the CI alone and bimodal conditions were closely aligned. The bottom four rows of Figure 5 illustrate CI ear dominance in the LBA group, where four CV syllables (/na/, /fa/, /va/, and /ʃa/) show this pattern. For instance, when /ʃa/ (the first row from the bottom) is the target, it is often confused with /ba/, /fa/, and /sa/ in the HA ear, while no major confusions were observed in either the CI alone or bimodal conditions, again resulting in similar perception scores across those two conditions. The remaining three CV syllables in this group showed similar patterns of confusion between the CI alone and bimodal conditions. These findings support the presence of CI ear dominance (i.e., better ear dominance) as one of the underlying mechanisms of bimodal hearing.

## 4 Discussion

The present study identified key differences in perceptual confusion between bimodal users who received greater bimodal advantage (GBA group) in consonant recognition and those with less bimodal advantage (LBA group). We aimed to answer two questions. The first question was which consonant contributed to each group. We found that the GBA group (*n* = 8) received a significant bimodal advantage from six CV syllables (/ga/, /ta/, /fa/, /ʃa/, /za/, and /ʤa/) and experienced no significant bimodal disadvantage. In contrast, the LBA group (*n* = 13) received a significant bimodal advantage from two CV syllables (/ga/ and /za/) and experienced a significant bimodal disadvantage from /ma/. Dichotomization of the two groups stemmed from significant differences in bimodal advantage from three CV syllables (/ta/, /za/, and /ʤa/). For the second question, concerning differences in confusion patterns, we found that confusion patterns were very different between groups in terms of the three listening strategies: spectral integration, interference, and CI-ear dominance. Spectral integration occurred on seven CV syllables (/ga/, /ma/, /fa/, /ʃa/, /va/, /za/, and /ʤa/) for the GBA group and on two CV syllables (/ga/ and /za/) for the LBA group. Spectral interference occurred on one CV syllable (/na/) for the GBA group and on eight CV syllables (/ba/, /da/, /pa/, /ta/, /ka/, /ma/, /sa/, and /ʤa/) for the LBA group. CI-ear dominance was observed from six CV syllables (/ba/, /da/, /pa/, /ta/, /ka/, and /sa/) for the GBA group and four CV syllables (/na/, /fa/, /ʃa/, and /va/) for the LBA group. These detailed analyses are necessary to gain a better understanding of why certain bimodal users receive the advantages and others do not. Raw confusion matrices are provided in the Supplementary material.

### 4.1 Bimodal advantage versus bimodal absolute performance

In this study, we calculated the bimodal advantage by taking the differences between performances with bimodal listening conditions and with the better ear alone. So, bimodal advantage is a derived quantity. In Figure 2, mean differences in bimodal advantage between the GBA and LBA groups are significant, but the mean performances with bimodal listening conditions between the groups are not. The derived bimodal advantage can mislead us to believe that the GBA group outperformed the LBA group, particularly in CI alone and bimodal listening conditions, which is not true. Instead, a larger bimodal advantage arises because of lower absolute CI alone performance (37.4, 45.9, and 59.8% for the GBA group versus 51.3, 57.8, and 66.2% for the LBA group at 5 dB, 10 dB, and quiet, respectively). Based on this result, some think that absolute bimodal performance should be used as an outcome measure in bimodal research rather than the derived bimodal advantage. However, we wanted to know how the GBA group received a significant bimodal advantage and why the LBA group did not, even though HA alone performances are similar between the two groups. We speculate that for the GBA group, each ear provides valuable consonant cues and integrates them, which results in greater bimodal advantage. In contrast, for the LBA group, the CI ear alone provides sufficient perceptual cues for consonant recognition, but integration occurs less actively. If CI alone performance is the sole defining factor for bimodal advantage, then a method can be developed to predict bimodal advantage in speech perception. That is, if CI alone performance is higher, the bimodal advantage can be expected to be less.

In terms of potential reasons for differences in CI alone performance between the two groups, we found similar PTA, age, etiology, and age at bimodal use between the two groups, based on Tables 1, 2. However, years of prior use of HA for the CI ear are different (23.0 for the GBA group vs. 16.3 for the LBA group), meaning that the LBA group received CI approximately 7 years earlier than the GBA group. Greater experience with CI in the LBA group helps the auditory system become more habituated to electric stimulations and spectral mismatch between mapped frequencies and tonotopic organization (Mok et al., 2007). Thus, better adaptation to electric stimulations may result in higher performance in CI ear alone conditions for the LBA group than for the GBA group. Other factors could be associated with post-implantation changes, such as the number and capability of surviving auditory nerve fibers and electrode placement, contributing to differences in CI alone performance (Teoh et al., 2003) across the two groups.

### 4.2 Comparisons in confusion matrices between GBA and LBA groups

The analysis of the confusion patterns between the GBA and LBA groups showed how the two groups performed under different listening conditions. For the GBA group, the confusion matrices reflected a more integrated response between the two ears, suggesting that they could use both devices’ inputs in a complementary way to process speech more accurately. The GBA group may combine low- and high-frequency information for 13 out of 14 CV syllables (except for /ka/). This integration helped them resolve potential errors seen when listening with just one device. As Yoon and Morgan (2022) explained, the integration of spectral information across ears is crucial for consonant recognition, even when significant spectral information is missing. They found that the ability to combine low-frequency information from one ear and high-frequency information from the other ear greatly enhanced speech perception, especially in difficult listening conditions. Zhang et al. (2020) found that bimodal hearing can significantly improve tone recognition, particularly in noisy environments. The study highlighted that adding acoustic information from the HA helps reduce confusion between similar sounds. This supports the idea that combining inputs from both ears can lead to clearer auditory perceptions, similar to the improvements seen in the GBA group. Yoon et al. (2015) highlighted that bimodal hearing benefits are closely linked to the performance differences between the CI and HA. The results indicated that the greater the similarity in performance between the two devices, the more significant the benefit in speech recognition, particularly in environments with varying levels of background noise. This suggests that effective integration of both devices’ inputs is crucial in maximizing the listener’s ability to process speech accurately.

On the other hand, the LBA group demonstrated a different pattern. Although they still benefited from bimodal hearing, their integration of both ears was less than that of the GBA group. In this group, only a few CV syllables showed clear spectral integration (5 out of 14 consonants), indicating that they were not as effective at combining information from both devices. This difference suggests that the CI ear alone may provide sufficient information for consonant recognition in many cases, leaving the HA ear’s contribution less important. This could be due to a higher degree of reliance on the CI ear, a phenomenon known as CI-ear dominance. Better ear dominance may explain these results in the LBA group as well, which was discussed below in E. CI-ear dominance. According to Kong and Braida (2011), bimodal hearing users benefit from complementary cues, especially for vowels, where the HA predominantly provides F1 (low-frequency) information and the CI provides more F2 (high-frequency) information. However, it is essential to note that not all CI listeners experience significant bimodal benefits, particularly when the cues from both devices overlap or are redundant. In line with this, Svirsky et al. (2020) found that the benefit of bimodal hearing can be influenced by the quality of the signal provided by each ear. In some cases, the CI provides a significantly higher quality signal than the acoustically aided ear, leading to a preference for the CI signal. This could explain why certain listeners may rely more on CI, neglecting the acoustic input from the non-implanted ear, affecting overall speech perception performance. Reiss et al. (2014) observed that bimodal cochlear implant users often experience a mismatch between the acoustic and electric signals from their hearing devices, leading to abnormal binaural spectral integration. This mismatch can cause speech perception interference, as mismatched spectral information from both ears is fused, sometimes resulting in pitch averaging and loss of individual spectral cues, which is consistent with the increased confusion seen in the LBA group. When comparing the two groups, the GBA group showed a more balanced contribution from both ears, resulting in better overall perception and fewer confusions in their consonant recognition. In contrast, the LBA group experienced more confusion with the same CV syllables, particularly in noisy conditions. This may be connected to their inability to integrate the acoustic and electric information as effectively, and the performance is more heavily influenced by the CI alone, or even leading to interference. This pattern highlights a key difference between the groups in how they process spectral information and resolve auditory confusion. These differences in the ability to combine and integrate information from both ears may explain why some bimodal users experience more significant advantages than others.

### 4.3 Spectral integration

Under the bimodal listening conditions, the transmission of richer spectral information is expected compared to CI alone because low-frequency acoustic information via an HA ear is available and integrates them with temporal envelope cues in the CI ear (Happel et al., 2010; Spehar et al., 2008). However, the present study showed different integration abilities between groups. Seven CV syllables (/ga/, /ma/, fa/, /ʃa/, /va/, /za, and /ʤa/) are in the spectral integration category for the GBA group (the top seven rows of Figure 3), but only two CV syllables (/ga/ and /za/) for the LBA group (the bottom two rows of Figure 3). The underlying mechanism for integration is not well understood. One potential explanation for the group differences is that spectral integration depends on the ability to integrate widely separated important spectral cues across ears, which is well documented in NH studies (Fox et al., 2011; Grose et al., 2016; Hall 3rd et al., 2008). That is, the GBA group may have a better ability to integrate two widely separated formant frequencies (e.g., F1 = 300 Hz in the HA ear and F2 = 2000 Hz in the CI ear) for consonant perception. In contrast, the LBA group may need more closely separated formant frequencies (e.g., F1 = 300 Hz in the HA ear and F2 = 900 Hz in the CI ear). Our pilot data support this concept; spectral integration depends on frequency differences between the first two formants for vowel recognition (Jaisinghani and Yoon, 2025).

Our data can also be explained by complementary and redundant cue integration. The subjects in the GBA group may have better “complementary cue integration,” which is defined as the fact that information provided by a HA ear and a CI ear is relatively different but complementary to each other. Yoon et al. (2015) claimed a similar theory by showing that bimodal advantage depended on differences in unilateral performance. That is, individuals who had smaller performance differences between the HA ear and CI ear received a greater bimodal advantage, while those with larger differences in unilateral performance received a lesser bimodal advantage. It is unclear whether smaller differences in unilateral performances indicate complementary cues to each other or not, but it does indicate that complementary information is available to integrate them and provide much richer information to the auditory system. For the LBA group, “redundant cue integration” may occur, in which CI alone provides all necessary information, and HA alone provides somewhat similar cues to the CI alone provided, so not much new information is provided. Two previous studies support this concept (Ma et al., 2016; Yoon et al., 2015). Bimodal advantage in speech perception is absent when the CI ear supports a superior level of monaural performance. These results suggest that information detected and processed by the CI ear is sufficient, and information provided by the HA ear is similar to that of the CI ear, which is less utilized for integration.

### 4.4 Spectral interference

Spectral interference is common in bilateral CI users (Laszig et al., 2004), individuals with bilateral hearing loss (McArdle et al., 2012), and the elderly population with both normal hearing and hearing loss (Mussoi and Bentler, 2017). In this study, spectral interference refers to a decrease in speech perception caused by conflicting auditory cues from the CI and HA. This interference occurs when the combined auditory input increases phonetic competition, resulting in more confusion and poorer speech recognition compared to using either ear alone. The GBA group had only the CV syllable /na/ in this category, while the LBA group had eight CV syllables (/ba/, /da/, /pa/, /ta/, /ka/, /ma/, /sa/, and /ʤa/). These results suggest that the integration of electric and acoustic stimulation is more interfered by certain factors in the LBA group than in the GBA group. Two potential factors for spectral interference are discussed below: simultaneous masking and temporal mismatch between the CI ear and HA ear.

Psychoacoustic simultaneous masking studies have shown a significantly elevated detection threshold of acoustic probe tones when an electric masker was simultaneously presented, and vice versa (Imsiecke et al., 2020a; Krüger et al., 2017). The simultaneous masking can limit the benefits of speech perception (Imsiecke et al., 2020b). These studies tested Electric Acoustic Stimulation (EAS) users who receive combined electric and acoustic stimulation in the same ear. They used an acoustic probe tone and an electric pulse train masker for electric masking and vice versa for acoustic masking when the probe signal and masker were presented simultaneously. One common finding in both Krüger et al. (2017) and Imsiecke et al. (2020a) is that a significant elevation of detection thresholds occurred over a wide electric-acoustic frequency difference, the distance between electric and acoustic stimulation in octaves of the place frequency. That is, a 1,000-Hz acoustic masker can elevate the detection threshold of a 4,000-Hz electric probe signal, and vice versa. These results suggest that bimodal hearing can interfere with either electric or acoustic signals in a very broad frequency range, leading to spectral interference. Imsiecke et al. (2020a) also observed reduced cortical responses in EAS users under both electric and acoustic maskers. This finding is supported by numerous animal studies, reporting that electric-acoustic interaction effects can result in reduced physiological responses in the cochlear nerve and the inferior colliculus (McAnally et al., 1997; Nourski et al., 2007; Stronks et al., 2010). Imsiecke et al. (2020b) measured the effect of this masking on speech perception in noise with EAS users. A larger benefit in speech perception was observed with a new frequency map, which was adjusted based on masking thresholds, and masking was significantly inversely correlated to the speech reception performance (Imsiecke et al., 2020b). Fowler et al. (2016) reported vowel interference in bimodal users who had better residual hearing (< 60 dB HL at frequencies below 750 Hz) in the HA ear. Bimodal users received improved vowel recognition when low-to-mid (approximately 440–982 Hz) frequencies in the CI ear were removed. Removing mid-frequency information processed by the CI ear may reduce the masking effect, consequently reducing spectral interference. Based on these detrimental effects of the masking, our subjects in the LBA group may experience significant masking effects, making spectral contents smeared and consequently spectral integration difficult. Spectral smearing can hinder speech recognition by blurring essential spectral details. Boothroyd et al. (1996) demonstrated that spectral smearing reduces access to formant-based spectral cues, especially those conveying consonant place information, resulting in poorer phoneme recognition. Similarly, Nittrouer et al. (2015) found that simulating broadened auditory filters to mimic spectral smearing significantly degraded phoneme recognition in noise. Under this listening situation, the subjects should have more confusion with the bimodal listening condition than with CI alone, leading to spectral interference. Further studies are needed to confirm a correlation between masking and interference.

Another factor that potentially causes spectral interference is a temporal mismatch across ears – a processing time delay between CI and HA. A significant temporal mismatch in bilateral CI users is common and results in a consistent lateralization bias (Williges et al., 2018). The average temporal mismatch between CI and HA is 5.9 ms ± 1.6 standard deviation, assessed with 167 HA models (Angermeier et al., 2021; Med-EL, 2023; Zirn et al., 2019; Zirn et al., 2015). Research showed that speech perception (Stone and Moore, 2003) and sound localization (Angermeier et al., 2021; Zirn et al., 2019) are not negatively affected if the temporal mismatch between the two devices is within 10 to 15 ms. However, cortical auditory evoked potentials in bimodal users were significantly decreased when the temporal mismatch was 4.3 ± 10.3 ms, and the temporal mismatch that led to the greatest amplitude of the N1 peak was 4.6 ± 10.3 ms (Dolhopiatenko et al., 2024). These results suggest that spectral interference can occur if the temporal mismatch is greater than 5 ms. As the processing time for HA and CI is not measured from our subjects, the temporal mismatch is unknown for each group, and the group difference is also unknown. Unlike the temporal mismatches originating from the devices, it should be noted that the neuronal processing time for HA ear and CI ear may also be different due to different degrees of auditory deprivation. CI ear (severe-to-profound hearing loss) typically experiences more auditory deprivation than HA ear (mild-to-severe hearing loss), leading to delayed access to information in the cortex (Polonenko et al., 2018). Adaptation to electric stimulation can also affect neuronal processing delay in the CI ear (Pérez-González and Malmierca, 2014). Speech perception in simulated single-sided CI had a modest reduction in the range of 0.5–12 ms (Wess et al., 2017). After temporal mismatch compensation, an acute improvement in sound localization in bimodal users is observed (Angermeier et al., 2023). Similarly, increased N1 amplitude was evident when the temporal mismatch was compensated (Dolhopiatenko et al., 2024). These results suggest that compensating for the temporal mismatch between electric and acoustic stimulation appears crucial to enhancing bimodal speech perception. The compensation for temporal mismatch can be done by adding zeros to the signal that will be presented to the ear with faster processing time.

### 4.5 CI-ear dominance

In CI-ear dominance, input from the CI is mainly processed and perceived, whereas input from the HA contributes less to perception (Reiss et al., 2016). In the present study, six CV syllables (/ba/, /da/, /pa/, ta/, /ka/, and /sa/) were in this category for the GBA group (top six rows of Figure 5), while four CV syllables (/na/, /fa/, /ʃa/, and /va/) were for the LBA group (bottom four rows of Figure 5). CI-ear dominance primarily occurred in the LBA group, showing that the mean difference between bimodal and CI alone listening conditions is four percentage points (range of −1.3 to 6.0 except for S21: 33.1), compared to 12.9 percentage points (range of 8.1 to 31.2) for the GBA group. It should be noted again that the LBA group shows more CI-ear dominance than the GBA group because, as discussed in section A. Bimodal advantage versus bimodal performance above, because a little bimodal advantage arises for the LBA group because of higher absolute CI alone performance (51.3, 57.8, and 66.2% for the LBA group versus 37.4, 45.9, and 59.8% for the GBA group at 5 dB, 10 dB, and quiet, respectively). However, ear dominance exists as a bimodal hearing phenomenon. Reiss et al. (2014) observed an ear dominance in a pitch fusion study with bimodal users. Oh and Reiss (2020) found that bimodal users experienced a pitch dominance by lower pitch when two tones were farther apart, which is also true in bilateral HA users (Oh and Reiss, 2017). It is theoretically true that under the CI-ear dominance, dichotic spectral integration is less affected by the HA ear, regardless of the degree of residual hearing. Further research is needed to explore how ear dominance affects pitch fusion and CI-ear dominance in speech perception.

### 4.6 Limitations

There are several limitations in the study. First, a small sample size can weaken the statistical power, particularly for the GBA group (*n* = 8). However, each CV syllable was presented ten to 30 times at each SNR in random order per listening condition. This procedure generated a row sum of 80 to 240 in the confusion matrices for each CV syllable with a sample size of eight, which is sufficient to observe reliable confusion patterns of individual consonants (Allen, 2005; Miller and Nicely, 1955). Second, HA frequency compression features can influence spectral processing for bimodal users. Perreau et al. (2013) showed a negative effect of both standard and adaptive frequency compressions on speech perception with bimodal hearing. Veugen et al. (2016) reported that the automatic gain control (AGC)-matched HA outperformed the HA with a standard AGC in speech understanding in noise tasks in bimodal users. Third, the fitting procedures and loudness balancing could be an issue. Two studies compared speech perception with three HA fitting procedures and two different loudness balancing approaches in bimodal users (Veugen et al., 2016; Vroegop et al., 2019). However, they found no differences in the provided gain or bimodal performance for the different HA fittings. Finally, although our study demonstrated a concept of integration, interference, and CI ear dominance, it was not specifically designed to test how certain sound features (e.g., F1 and F2 formants) combine across the ears in real-life bimodal users. The stimuli we used were not chosen in a way that would allow us to clearly see how each ear processes different parts of the stimuli. In future studies, it would be helpful to design tests that focus more closely on how the brain puts together stimuli from both ears, especially when it comes to understanding vowels or speech sounds.

## Data Availability

The datasets presented in this study can be found in online repositories. The names of the repository/repositories and accession number(s) can be found in the article/Supplementary material.

## References

[ref1] Allen J. B. (2005). Consonant recognition and the articulation index. J. Acoust. Soc. Am. 117, 2212–2223. doi: 10.1121/1.1856231, 15898662

[ref2] Angermeier J. Hemmert W. Zirn S. (2021). Sound localization bias and error in bimodal listeners improve instantaneously when the device delay mismatch is reduced. Trends Hear. 25:23312165211016165. doi: 10.1177/23312165211016165, 34057366 PMC8182625

[ref3] Angermeier J. Hemmert W. Zirn S. (2023). Clinical feasibility and familiarization effects of device delay mismatch compensation in bimodal CI/HA users. Trends Hearing 27:23312165231171987. doi: 10.1177/23312165231171987, 37194477 PMC10196534

[ref4] Bernstein J. G. W. Stakhovskaya O. A. Jensen K. K. Goupell M. J. (2019). Acoustic hearing can interfere with single-sided deafness cochlear-implant speech perception. Ear Hear. 41, 747–761. doi: 10.1097/aud.0000000000000805, 31584504 PMC7117997

[ref5] Boersma P. (2001). Praat, a system for doing phonetics by computer. Glot. Int. 5, 341–345. Available online at: https://dare.uva.nl/search?metis.record.id=200596

[ref6] Boothroyd A. Mulhearn B. Gong J. Ostroff J. (1996). Effects of spectral smearing on phoneme and word recognition. J. Acoust. Soc. Am. 100, 1807–1818. doi: 10.1121/1.416000, 8817914

[ref7] Buchman C. A. Herzog J. A. McJunkin J. L. Wick C. C. Durakovic N. Firszt J. B. . (2020). Assessment of speech understanding after cochlear implantation in adult hearing aid users: a nonrandomized controlled trial. JAMA Otolaryngol. Head Neck Surg. 146, 916–924. doi: 10.1001/jamaoto.2020.1584, 32857113 PMC7453346

[ref8] Chatterjee M. Peng S.-C. (2008). Processing F0 with cochlear implants: modulation frequency discrimination and speech intonation recognition. Hear. Res. 235, 143–156. doi: 10.1016/j.heares.2007.11.004, 18093766 PMC2237883

[ref9] Cox R. M. (1995). Using loudness data for hearing aid selection: the IHAFF approach. Hear. J. 48, 39–44.

[ref10] Dolhopiatenko H. Segovia-Martinez M. Nogueira W. (2024). The temporal mismatch across listening sides affects cortical auditory evoked responses in normal hearing listeners and cochlear implant users with contralateral acoustic hearing. Hear. Res. 451:109088. doi: 10.1016/j.heares.2024.109088, 39032483

[ref11] Dorman M. F. Gifford R. H. Spahr A. J. McKarns S. A. (2008). The benefits of combining acoustic and electric stimulation for the recognition of speech, voice and melodies. Audiol. Neurootol. 13, 105–112. doi: 10.1159/000111782, 18057874 PMC3559130

[ref12] Dubno J. R. Dorman M. F. (1987). Effects of spectral flattening on vowel identification. J. Acoust. Soc. Am. 82, 1503–1511. doi: 10.1121/1.395194, 3693692

[ref13] Fousek P. Svojanovsky P. Grezl F. Hermansky H. (2004). New nonsense syllables database: Analyses and preliminary ASR experiments. The international conference on spoken language processing. Available online at: https://www.isca-archive.org/interspeech_2004/fousek04_interspeech.pdf

[ref14] Fowler J. R. Eggleston J. L. Reavis K. M. McMillan G. P. Reiss L. A. (2016). Effects of removing low-frequency electric information on speech perception with bimodal hearing. J Speech Language Hearing Research 59, 99–109. doi: 10.1044/2015_jslhr-h-15-0247, 26535803 PMC4862739

[ref15] Fox R. A. Jacewicz E. Chang C. Y. (2011). Auditory spectral integration in the perception of static vowels. J Speech Language Hearing Research 54, 1667–1681. doi: 10.1044/1092-4388(2011/09-0279), 21862680 PMC4486011

[ref16] Fraysse B. Macias A. R. Sterkers O. Burdo S. Ramsden R. Deguine O. . (2006). Residual hearing conservation and electroacoustic stimulation with the nucleus 24 contour advance cochlear implant. Otol. Neurotol. 27, 624–633. doi: 10.1097/01.mao.0000226289.04048.0f, 16868510

[ref17] Fu Q.-J. Galvin J. J. III (2008). Maximizing cochlear implant patients’ performance with advanced speech training procedures. Hear. Res. 242, 198–208. doi: 10.1016/j.heares.2007.11.010, 18295992 PMC2603139

[ref18] Fu Q.-J. Galvin J. J. III Wang X. (2017a). Effect of carrier bandwidth on integration of simulations of acoustic and electric hearing within or across ears. J. Acoust. Soc. Am. 142:EL561-EL566. doi: 10.1121/1.5017530, 29289073 PMC6909988

[ref19] Fu Q.-J. Galvin J. J. Wang X. (2017b). Integration of acoustic and electric hearing is better in the same ear than across ears. Sci. Rep. 7, 1–9. doi: 10.1038/s41598-017-12298-3 28970567 PMC5624923

[ref20] Gifford R. H. Dorman M. F. (2019). Bimodal hearing or bilateral Cochlear implants? Ask the patient. Ear Hear. 40, 501–516. doi: 10.1097/aud.0000000000000657, 30285977 PMC6447482

[ref21] Gifford R. H. Dorman M. F. McKarns S. A. Spahr A. J. (2007). Combined electric and contralateral acoustic hearing: word and sentence recognition with bimodal hearing. J Speech Language Hearing Research 50, 835–843. doi: 10.1044/1092-4388(2007/058), 17675589 PMC2441834

[ref22] Gifford R. H. Dorman M. F. Skarzynski H. Lorens A. Polak M. Driscoll C. L. . (2013). Cochlear implantation with hearing preservation yields significant benefit for speech recognition in complex listening environments. Ear Hear. 34, 413–425. doi: 10.1097/AUD.0b013e31827e8163, 23446225 PMC3742689

[ref23] Goupell M. J. Stakhovskaya O. A. Bernstein J. G. W. (2018). Contralateral interference caused by binaurally presented competing speech in adult bilateral Cochlear-implant users. Ear Hear. 39, 110–123. doi: 10.1097/aud.0000000000000470, 28787316 PMC5741461

[ref24] Grose J. H. Porter H. L. Buss E. (2016). Aging and spectro-temporal integration of speech. Trends Hear. 20, 1–11. doi: 10.1177/2331216516670388, 27742880 PMC5068923

[ref25] Hall J. W. 3rd Buss E. Grose J. H. (2008). Spectral integration of speech bands in normal-hearing and hearing-impaired listeners. J. Acoust. Soc. Am. 124, 1105–1115. doi: 10.1121/1.2940582 18681600 PMC2633714

[ref26] Happel M. F. Jeschke M. Ohl F. W. (2010). Spectral integration in primary auditory cortex attributable to temporally precise convergence of thalamocortical and intracortical input. J. Neurosci. 30, 11114–11127. doi: 10.1523/jneurosci.0689-10.2010, 20720119 PMC6633479

[ref27] Hayden R. E. (1950). The relative frequency of phonemes in general-American English. Word 6, 217–223. doi: 10.1080/00437956.1950.11659381

[ref28] Hertrich I. Mathiak K. Lutzenberger W. Ackermann H. J. N. (2002). Hemispheric lateralization of the processing of consonant-vowel syllables (formant transitions): effects of stimulus characteristics and attentional demands on evoked magnetic fields. Neuropsychologia 40, 1902–1917. doi: 10.1016/s0028-3932(02)00063-5, 12207989

[ref29] Imsiecke M. Büchner A. Lenarz T. Nogueira W. (2020a). Psychoacoustic and electrophysiological electric-acoustic interaction effects in cochlear implant users with ipsilateral residual hearing. Hear. Res. 386:107873. doi: 10.1016/j.heares.2019.107873, 31884220

[ref30] Imsiecke M. Krüger B. Büchner A. Lenarz T. Nogueira W. (2020b). Interaction between electric and acoustic stimulation influences speech perception in ipsilateral EAS users. Ear Hear. 41, 868–882. doi: 10.1097/aud.0000000000000807, 31592902 PMC7676483

[ref31] Incerti P. V. Ching T. Y. Hill A. (2011). Consonant perception by adults with bimodal fitting. Semin. Hear. 32, 090–102. doi: 10.1055/s-0031-1271950

[ref32] Jaisinghani P. Yoon Y. S. (2025). Effects of F1-F2 frequency spacing on spectral integration in combined electric and acoustic stimulation. J. Speech Lang. Hear. Res. 68, 792–807. doi: 10.1044/2024_jslhr-24-00273, 39787532 PMC11842090

[ref33] Ketten D. R. Skinner M. W. Wang G. Vannier M. W. Gates G. A. Neely J. G. (1998). In vivo measures of cochlear length and insertion depth of nucleus cochlear implant electrode arrays. Ann. Otol. Rhinol. Laryngol. 175, 1–16. 9826942

[ref34] Kiefer J. Pok M. Adunka O. Sturzebecher E. Baumgartner W. Schmidt M. . (2005). Combined electric and acoustic stimulation of the auditory system: results of a clinical study. Audiol. Neurootol. 10, 134–144. doi: 10.1159/000084023, 15724084

[ref35] Kong Y. Y. Braida L. D. (2011). Cross-frequency integration for consonant and vowel identification in bimodal hearing. J. Speech Lang. Hear. Res. 54, 959–980. doi: 10.1044/1092-4388(2010/10-0197), 21060139 PMC3107368

[ref36] Krüger B. Büchner A. Nogueira W. (2017). Simultaneous masking between electric and acoustic stimulation in cochlear implant users with residual low-frequency hearing. Hear. Res. 353, 185–196. doi: 10.1016/j.heares.2017.06.014, 28688755

[ref37] Laszig R. Aschendorff A. Stecker M. Müller-Deile J. Maune S. Dillier N. . (2004). Benefits of bilateral electrical stimulation with the nucleus cochlear implant in adults: 6-month postoperative results. Otol. Neurotol. 25, 958–968. doi: 10.1097/00129492-200411000-00016, 15547426

[ref38] Ma N. Morris S. Kitterick P. T. (2016). Benefits to speech perception in noise from the binaural integration of electric and acoustic signals in simulated unilateral deafness. Ear Hear. 37, 248–259. doi: 10.1097/aud.0000000000000252, 27116049 PMC4847646

[ref39] McAnally K. I. Brown M. Clark G. M. (1997). Estimating mechanical responses to pulsatile electrical stimulation of the cochlea. Hear. Res. 106, 146–153. doi: 10.1016/s0378-5955(97)00012-9, 9112114

[ref40] McArdle R. A. Killion M. Mennite M. A. Chisolm T. H. (2012). Are two ears not better than one? J. Am. Acad. Audiol. 23, 171–181. doi: 10.3766/jaaa.23.3.4, 22436115

[ref41] Med-EL 2023 Timing Settings for Hearing Aids: Bimodal Synchronization in MAESTRO. Available online at: https://www.medel.pro/online-resources/timing-settings-for-hearing-aids

[ref42] Miller G. A. Nicely P. E. (1955). An analysis of perceptual confusions among some English consonants. J. Acoust. Soc. Am. 27, 338–352. doi: 10.1121/1.1907526

[ref43] Mok M. Galvin K. L. Dowell R. C. McKay C. M. (2007). Spatial unmasking and binaural advantage for children with normal hearing, a cochlear implant and a hearing aid, and bilateral implants. Audiol. Neurootol. 12, 295–306. doi: 10.1159/000103210, 17536198

[ref44] Mok M. Galvin K. L. Dowell R. C. McKay C. M. (2010). Speech perception benefit for children with a cochlear implant and a hearing aid in opposite ears and children with bilateral cochlear implants. Audiol. Neurootol. 15, 44–56. doi: 10.1159/000219487, 19468210

[ref45] Mok M. Grayden D. Dowell R. C. Lawrence D. (2006). Speech perception for adults who use hearing aids in conjunction with cochlear implants in opposite ears. J. Speech Lang. Hear. Res. 49, 338–351. doi: 10.1044/1092-4388(2006/027), 16671848

[ref46] Mussoi B. S. Bentler R. A. (2017). Binaural interference and the effects of age and hearing loss. J. Am. Acad. Audiol. 28, 5–13. doi: 10.3766/jaaa.15011, 28054908

[ref47] Neuman A. C. Waltzman S. B. Shapiro W. H. Neukam J. D. Zeman A. M. Svirsky M. A. (2017). Self-reported usage, functional benefit, and Audiologic characteristics of Cochlear implant patients who use a contralateral hearing aid. Trends Hearing 21:2331216517699530. doi: 10.1177/2331216517699530, 28351216 PMC5435367

[ref48] Neuman A. C. Zeman A. Neukam J. Wang B. Svirsky M. A. (2019). The effect of hearing aid bandwidth and configuration of hearing loss on bimodal speech recognition in Cochlear implant users. Ear Hear. 40, 621–635. doi: 10.1097/aud.0000000000000638, 30067559 PMC6355393

[ref49] Nittrouer S. Tarr E. Wucinich T. Moberly A. C. Lowenstein J. H. (2015). Measuring the effects of spectral smearing and enhancement on speech recognition in noise for adults and children. J. Acoust. Soc. Am. 137, 2004–2014. doi: 10.1121/1.4916203, 25920851 PMC4417020

[ref50] Nourski K. V. Abbas P. J. Miller C. A. Robinson B. K. Jeng F. C. (2007). Acoustic-electric interactions in the guinea pig auditory nerve: simultaneous and forward masking of the electrically evoked compound action potential. Hear. Res. 232, 87–103. doi: 10.1016/j.heares.2007.07.001, 17723284 PMC2048988

[ref51] Oh Y. Reiss L. J. (2017). Binaural pitch fusion: pitch averaging and dominance in hearing-impaired listeners with broad fusion. J. Acoust. Soc. Am. 142:780. doi: 10.1121/1.4997190 28863555 PMC5648564

[ref52] Oh Y. Reiss L. A. J. (2020). Binaural pitch fusion: binaural pitch averaging in Cochlear implant users with broad binaural fusion. Ear Hear. 41, 1450–1460. doi: 10.1097/aud.0000000000000866, 33136622 PMC7501189

[ref53] Pérez-González D. Malmierca M. S. (2014). Adaptation in the auditory system: an overview. Front. Integr. Neurosci. 8:19. doi: 10.3389/fnint.2014.00019, 24600361 PMC3931124

[ref54] Perreau A. E. Bentler R. A. Tyler R. S. (2013). The contribution of a frequency-compression hearing aid to contralateral cochlear implant performance. J. Am. Acad. Audiol. 24, 105–120. doi: 10.3766/jaaa.24.2.4, 23357804

[ref55] Phatak S. A. Yoon Y. S. Gooler D. M. Allen J. B. (2009). Consonant recognition loss in hearing impaired listeners. J. Acoust. Soc. Am. 126, 2683–2694. doi: 10.1121/1.3238257, 19894845 PMC2787079

[ref56] Pieper S. H. Hamze N. Brill S. Hochmuth S. Exter M. Polak M. . (2022). Considerations for fitting cochlear implants bimodally and to the single-sided deaf. Trends Hear. 26:23312165221108259. doi: 10.1177/23312165221108259, 35726211 PMC9218456

[ref57] Pillsbury H. C. 3rd Dillon M. T. Buchman C. A. Staecker H. Prentiss S. M. Ruckenstein M. J. . (2018). Multicenter US clinical trial with an electric-acoustic stimulation (EAS) system in adults: final outcomes. Otol. Neurotol. 39, 299–305. doi: 10.1097/mao.0000000000001691, 29342054 PMC5821485

[ref58] Polonenko M. J. Papsin B. C. Gordon K. A. (2018). Delayed access to bilateral input alters cortical organization in children with asymmetric hearing. NeuroImage Clinical 17, 415–425. doi: 10.1016/j.nicl.2017.10.036, 29159054 PMC5683809

[ref59] Reiss L. A. Eggleston J. L. Walker E. P. Oh Y. (2016). Two ears are not always better than one: mandatory vowel fusion across spectrally mismatched ears in hearing-impaired listeners. J. Assoc. Res. Otolaryngol. JARO 17, 341–356. doi: 10.1007/s10162-016-0570-z, 27220769 PMC4940290

[ref60] Reiss L. A. Fowler J. R. Hartling C. L. Oh Y. (2018). Binaural pitch fusion in bilateral cochlear implant users. Ear Hear. 39, 390–397. doi: 10.1097/aud.0000000000000497, 28945657 PMC5821581

[ref61] Reiss L. A. Ito R. A. Eggleston J. L. Wozny D. R. (2014). Abnormal binaural spectral integration in cochlear implant users. J. Assoc. Res. Otolaryngol. 15, 235–248. doi: 10.1007/s10162-013-0434-8, 24464088 PMC3946135

[ref62] Rødvik A. K. Tvete O. Torkildsen J. V. K. Wie O. B. Skaug I. Silvola J. T. (2019). Consonant and vowel confusions in well-performing children and adolescents with Cochlear implants, measured by a nonsense syllable repetition test. Front. Psychol. 10:1813. doi: 10.3389/fpsyg.2019.01813, 31474900 PMC6702790

[ref63] Sheffield S. W. Simha M. Jahn K. N. Gifford R. H. (2016). The effects of acoustic bandwidth on simulated bimodal benefit in children and adults with Normal hearing. Ear Hear. 37, 282–288. doi: 10.1097/aud.0000000000000281, 26901264 PMC4844770

[ref64] Skarzynski H. van de Heyning P. Agrawal S. Arauz S. L. Atlas M. Baumgartner W. . (2013). Towards a consensus on a hearing preservation classification system. Acta Otolaryngol. 133, 3–13. doi: 10.3109/00016489.2013.869059, 24328756

[ref65] Spehar B. P. Tye-Murray N. Sommers M. S. (2008). Intra- versus intermodal integration in young and older adults. J. Acoust. Soc. Am. 123, 2858–2866. doi: 10.1121/1.2890748, 18529201 PMC2811549

[ref66] Stone M. A. Moore B. C. (2003). Tolerable hearing aid delays. III. Effects on speech production and perception of across-frequency variation in delay. Ear Hear. 24, 175–183. doi: 10.1097/01.Aud.0000058106.68049.9c, 12677113

[ref67] Stronks H. C. Versnel H. Prijs V. F. Klis S. F. (2010). Suppression of the acoustically evoked auditory-nerve response by electrical stimulation in the cochlea of the guinea pig. Hear. Res. 259, 64–74. doi: 10.1016/j.heares.2009.10.004, 19840841

[ref68] Svirsky M. A. Neuman A. C. Neukam J. D. Lavender A. Miller M. K. Aaron K. A. . (2020). Speech perception changes in the acoustically aided, nonimplanted ear after cochlear implantation: a multicenter study. J. Clin. Med. 9:1758. doi: 10.3390/jcm9061758, 32517138 PMC7356938

[ref69] Teoh S. W. Neuburger H. S. Svirsky M. A. (2003). Acoustic and electrical pattern analysis of consonant perceptual cues used by Cochlear implant users. Audiology Neurotology 8, 269–285. doi: 10.1159/000072000, 12904682

[ref70] van Loon M. C. Smits C. Smit C. F. Hensen E. F. Merkus P. (2017). Cochlear implantation in adults with asymmetric hearing loss: benefits of bimodal stimulation. Otol. Neurotol. 38, e100–e106. doi: 10.1097/mao.0000000000001418, 28441230

[ref71] Veugen L. C. Chalupper J. Snik A. F. Opstal A. J. Mens L. H. (2016). Matching automatic gain control across devices in bimodal cochlear implant users. Ear Hear. 37, 260–270. doi: 10.1097/aud.0000000000000260, 26656192

[ref72] Veugen L. C. Chalupper J. Snik A. F. van Opstal A. J. Mens L. H. (2016). Frequency-dependent loudness balancing in bimodal cochlear implant users. Acta Otolaryngol. 136, 775–781. doi: 10.3109/00016489.2016.1155233, 26986743

[ref73] Vroegop J. L. Dingemanse J. G. van der Schroeff M. P. Goedegebure A. (2019). Comparing the effect of different hearing aid fitting methods in bimodal cochlear implant users. Am. J. Audiol. 28, 1–10. doi: 10.1044/2018_aja-18-0067, 30383163

[ref74] Wess J. M. Brungart D. S. Bernstein J. G. W. (2017). The effect of Interaural mismatches on contralateral unmasking with single-sided vocoders. Ear Hear. 38, 374–386. doi: 10.1097/aud.0000000000000374, 28002083

[ref75] Williges B. Jürgens T. Hu H. Dietz M. (2018). Coherent coding of enhanced interaural cues improves sound localization in noise with bilateral cochlear implants. Trends Hear. 22:2331216518781746. doi: 10.1177/2331216518781746, 29956589 PMC6048749

[ref76] Wongoutong C. (2024). The impact of neglecting feature scaling in k-means clustering. PLoS One 19:e0310839. doi: 10.1371/journal.pone.0310839, 39642177 PMC11623793

[ref77] Yoon Y. S. Li Y. Fu Q. J. (2012a). Speech recognition and acoustic features in combined electric and acoustic stimulation. J Speech Language Hearing Research 55, 105–124. doi: 10.1044/1092-4388(2011/10-0325), 22199183 PMC3288603

[ref78] Yoon Y. S. Morgan D. (2022). Dichotic spectral integration range for consonant recognition in listeners with normal hearing. Front. Psychol. 13, 1–14. doi: 10.3389/fpsyg.2022.1009463 PMC9633255 36337493

[ref79] Yoon Y. S. Riley B. Patel H. Frost A. Fillmore P. Gifford R. . (2019a). Enhancement of consonant recognition in bimodal and Normal hearing listeners. Ann. Otol. Rhinol. Laryngol. 128, 139s–145s. doi: 10.1177/0003489419832625, 31092038 PMC7174026

[ref80] Yoon Y. S. Shin Y. R. Gho J. S. Fu Q. J. (2015). Bimodal benefit depends on the performance difference between a cochlear implant and a hearing aid. Cochlear Implants Int. 16, 159–167. doi: 10.1179/1754762814y.0000000101, 25329752 PMC5847325

[ref81] Zhang T. Dorman M. F. Spahr A. J. (2010). Information from the voice fundamental frequency (F0) region accounts for the majority of the benefit when acoustic stimulation is added to electric stimulation. Ear Hear. 31, 63–69. doi: 10.1097/aud.0b013e3181b7190c, 20050394 PMC3684557

[ref82] Zhang H. Zhang J. Ding H. Zhang Y. (2020). Bimodal benefits for lexical tone recognition: an investigation on mandarin-speaking preschoolers with a cochlear implant and a contralateral hearing aid. Brain Sci. 10:238. doi: 10.3390/brainsci10040238, 32316466 PMC7226140

[ref83] Zhu A. Hua Z. Shi Y. Tang Y. Miao L. (2021). An improved K-means algorithm based on evidence distance. Entropy 23, 1–15. doi: 10.3390/e23111550, 34828248 PMC8625371

[ref84] Zirn S. Angermeier J. Arndt S. Aschendorff A. Wesarg T. (2019). Reducing the device delay mismatch can improve sound localization in bimodal Cochlear implant/hearing-aid users. Trends Hearing 23:2331216519843876. doi: 10.1177/2331216519843876, 31018790 PMC6484236

[ref85] Zirn S. Arndt S. Aschendorff A. Wesarg T. (2015). Interaural stimulation timing in single sided deaf cochlear implant users. Hear. Res. 328, 148–156. doi: 10.1016/j.heares.2015.08.010, 26302945

